# Mapping the adoption of artificial intelligence in urban mobility

**DOI:** 10.12688/openreseurope.23222.1

**Published:** 2026-03-10

**Authors:** Meng Cai

**Affiliations:** 1 Department of Civil and Environmental Engineering, TU Darmstadt, Darmstadt, 64287, Germany

**Keywords:** artificial intelligence, AI adoption, AI deployment, urban mobility, transportation, content analysis, Europe, empirical study

## Abstract

Urban transportation systems are undergoing a critical transformation driven by advances in artificial intelligence (AI). Despite many technical and theoretical discussions of AI technologies and a few case studies of AI implementation in selected cities, there remains a large gap in our knowledge about the landscape of the adoption of AI in urban mobility.

This study narrows this gap by addressing: where, when, how, and for which modes is AI being integrated into urban mobility across Europe? By text mining authoritative and publicly available data sources, i.e., EU Urban Mobility Observatory and official city websites, complemented by manual content analysis, this work documents the geographic distribution, temporal trends, use cases, and transportation modes of AI adoption.

As of September 2025, I identified 88 AI deployments across 73 cities in 21 European countries. It shows a clear acceleration in adoption after 2022, peaking in 2024 and remaining high in 2025. The deployments fall into eight broad categories: traffic monitoring and management; transport infrastructure condition assessment; parking management; improving bus and bike sharing systems; traffic control; autonomous and new mobility systems; data integration and digital twins; and public-facing digital services. In terms of transport mode, adoption is road-centric, with road applications accounting for 89% of recorded cases, while rail, air, waterborne, and cross-modal deployments are relatively rare. The compiled data is openly available online.

This work is among the first efforts to establish an empirical base for assessing the prevalence of AI in urban mobility. It aims to provide urban planners, policymakers, and researchers with a better understanding of current AI adoption practices in the European context.

## 1. Introduction

Urban mobility in Europe stands at the cusp of a transformative era as artificial intelligence (AI) and data infrastructures are reshaping transport services ( European Commission, 2025a; Jevinger et al., 2024). A review of academic literature indicates that transportation and mobility are among the sectors most significantly influenced by AI ( Herath & Mittal, 2022). The confluence of large-scale data availability, pressing urban challenges, and policy agendas has paved the way for AI applications, from traffic control optimization, shared autonomous vehicles, to computer-vision enabled road-user behavior detection ( Dilek & Dener, 2023; Jain & Pandey, 2025; Karolemeas et al., 2024; Ventura et al., 2025).

Yet despite a growing number of AI initiatives, the empirical landscape of AI adoption in urban mobility remains under-documented. Existing studies on smart cities and mobility often treat AI as one component of broader digital transformation and focus on its technical potential. The literature tends to describe what AI could do rather than what it actually does in real-world deployment. Recent research shows how AI can enhance routing, traffic flow prediction, and urban planning optimization by taking advantage of large mobility datasets ( Noaeen et al., 2022; Rocco di Torrepadula et al., 2024; Shaygan et al., 2022). For example, Noaeen et al. (2022) examined reinforcement learning in the network-scale traffic signal control and pointed out that many studies do not deal with or include real-world data, which challenges the validity of results. Research has also evaluated AI usage in road vehicle automation and smart traffic control, and noted challenges of trust, explainability, governance capacity, and data availability in real-world deployments ( Englund et al., 2021).

The few empirically grounded studies are often narrow in scope and rarely examine deployments beyond pilot sites. Large-scale empirical analyses across multiple cities and transportation modes are still rare. A case study of five cities on AI usage in Intelligent Transportation Systems found that AI can enhance traffic efficiency, reduce accidents, and support sustainable urban growth, but challenges around data quality, real-time processing, security, public acceptance, and privacy still need to be resolved ( Zemmouchi-Ghomari, 2025). An evaluation of 12 shared automated vehicle pilots in Europe found that shared robotaxis appear safe in closed environments but have more incidents in mixed traffic ( Debbaghi et al., 2025). The authors also call for standardized data collection processes and emphasize the need for baseline data for future deployments.

Taken together, these strands of research point to a critical gap: despite technical potential and proliferating pilots, the record of AI adoption in urban mobility remains fragmented and episodic. This work responds to this gap by constructing an open database of AI adoption in urban mobility systems across European cities. It documents four dimensions of AI adoption in Europe: 1) where has AI been deployed; 2) when deployments occurred; 3) how AI is integrated (i.e., application use cases); and 4) which transportation modes are involved (road, rail, air, waterborne, or cross modal).

In doing so, this project bridges between the theoretical discussions, technical analyses, and the grounding of AI adoption in urban contexts. The database provides an open resource that enables transport practitioners, researchers, and policymakers to monitor AI deployments, conduct comparative evaluations across place and time, and inform future modelling of AI’s evolving trend in urban mobility.

## 2. Methodology

I used automated keyword searches and manual content analysis to identify cases of AI adoption across Europe. More specifically, I first used the Google Custom Search Application Programming Interface to locate webpages on the EU Urban Mobility Observatory ( European Commission, 2025b) and on the official websites of 1448 European cities that mention the keyword “artificial intelligence.” These sources were chosen because they are both authoritative and openly available. The list of European city websites was adopted from Karam et al. (2025). To account for language differences, the keyword was translated into the language of each website. For example, for Hungarian websites, the search keyword was “mesterséges intelligencia.” In this step, I recorded the search date, title, short description, and link of each returned webpage. This step of data collection was conducted between late July to early September 2025. The Urban Mobility Observatory search returned 21 webpages, while the search across city websites returned a total of 13423 webpages mentioning AI.

Next, the title and short description of each webpage were manually reviewed to filter out webpages not related to transportation or mobility. Webpages deemed relevant were then examined further to determine whether they were truly about real-world AI deployment in urban mobility systems. Feasibility studies, plans, or discussions of potential AI implementation were not counted as actual adoption. When a webpage was considered relevant to AI adoption in urban mobility, the year of adoption, use case, deploying city, and transportation mode were recorded. When the year of adoption was not explicitly stated, the latest possible date, for example, the date when the news was published, was used as a proxy.

Finally, the identified AI adoption cases were transformed into JSON format and presented as an interactive online map ( https://caimeng2.github.io/UrbMobAI/). Each city with AI adoption was marked with a pin, and a slider bar allowed users to explore adoptions by year. Clicking on a pin reveals information about the use case, transportation mode, and the source, i.e., the link to the original webpage. In the spirit of open science, the online map is released under a Creative Commons Attribution 4.0 International License. Users may freely download the dataset for their own projects.

## 3. Results

**Figure 1.**
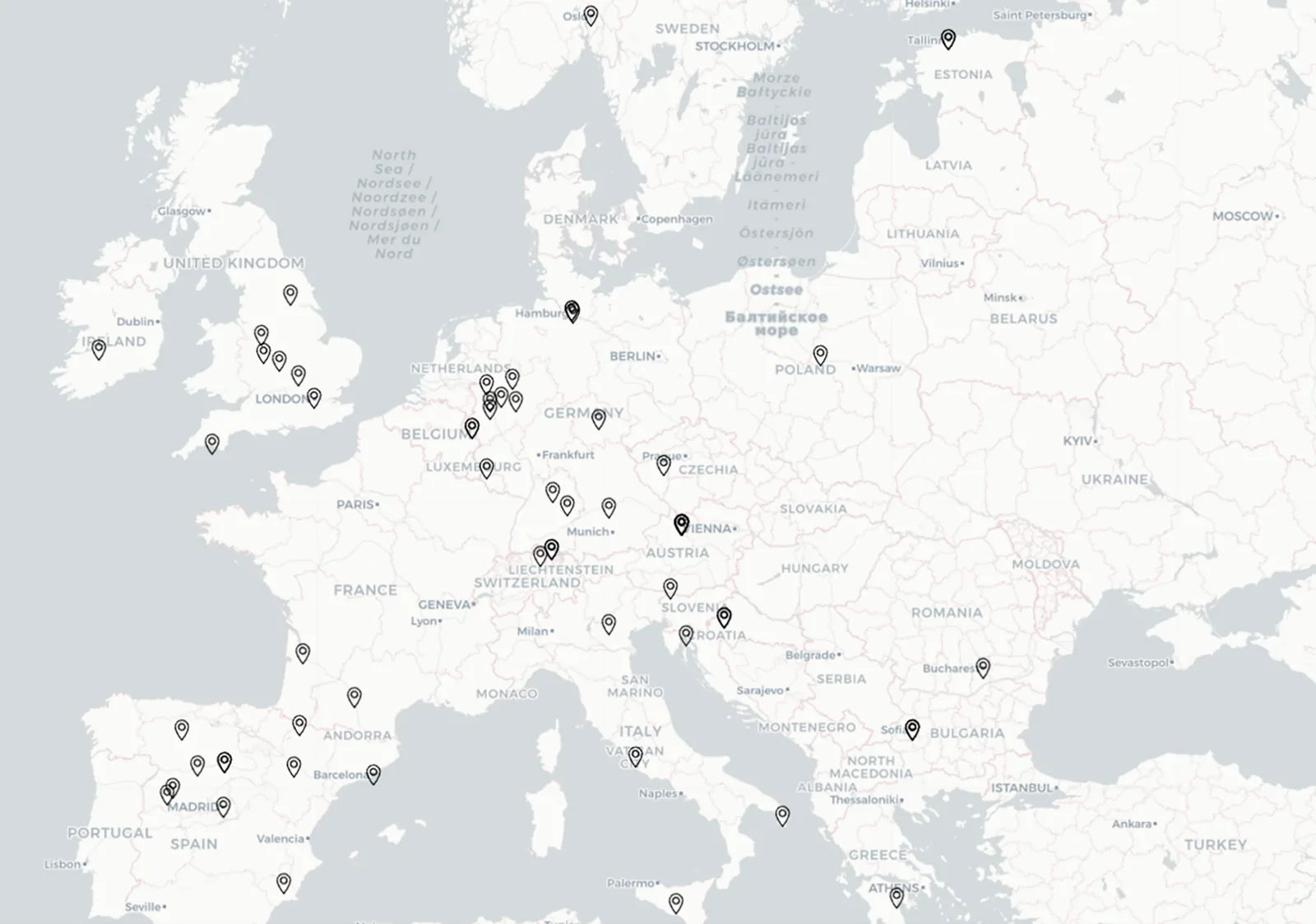
Screenshot of the interactive online map displaying AI adoption in Europe.

As of September 2025, this work documents a total of 88 AI deployments in urban mobility across 73 cities in 21 European countries ( Figure 1). The largest numbers of cases are found in Spain (18 cases), Germany (18 cases), the United Kingdom (7 cases), Austria (7 cases), and Italy (5 cases). Deployments are also recorded in Croatia (3 cases), Belgium (3 cases), Poland (3 cases), Russia (3 cases), Romania (3 cases), Bulgaria (3 cases), France (2 cases), Estonia (2 cases), Sweden (2 cases), Norway (2 cases), and Finland (2 cases), and in single cases in Greece, Ireland, Switzerland, the Netherlands, and Czechia.

**Figure 2.**
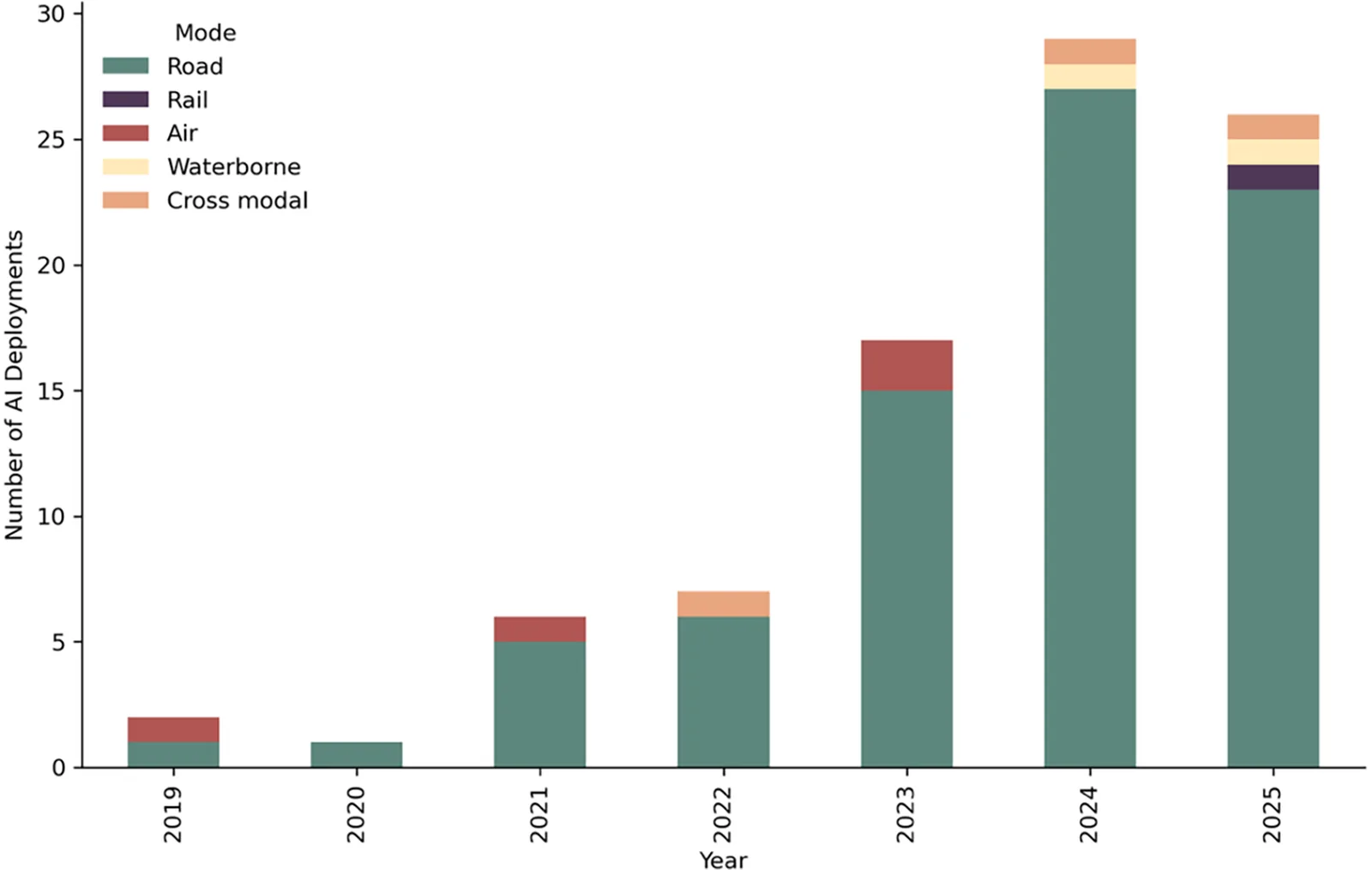
AI deployments in urban mobility by transportation mode over time.

The first recorded cases of AI adoption in urban mobility in Europe occurred in 2019 ( Figure 2). Identified AI deployments increased gradually from isolated cases between 2019 and 2022, followed by an expansion in 2023 (17 cases) and a peak in 2024 (29 cases). 26 deployments have been recorded in 2025 up to September.

The AI use cases fall into eight broad categories and cluster primarily around operational and infrastructural optimization ( Table 1). Traffic monitoring and management (n = 17) and transport infrastructure condition assessment (n = 16) are the most prevalent, followed by parking management (n = 12), traffic control (n = 10), and the improvement of bus and bike sharing systems (n = 10). Data integration and digital twin applications (n = 8), autonomous and new mobility systems (n = 8), and public-facing digital services (n = 7) appear less frequently.

**Table 1.** AI use cases identified in European cities.

AI use cases	Count	City of deployment
Traffic monitoring and management	17	Alba Iulia (Romania); Alicante (Spain); Almería (Spain); Bodø (Norway); Coventry (United Kingdom); Dos Hermanas (Spain); Estepona (Spain); Gdynia (Poland); Hamburg (Germany); London (United Kingdom); Plock (Poland); Plymouth (United Kingdom); Rimini (Italy); Sevilla (Spain); Sofia (Bulgaria); The Hague (Netherlands); Winterthur (Switzerland)
Transport infrastructure condition assessment	16	Duisburg (Germany); Düsseldorf (Germany); Herne (Germany); Konstanz (Germany); Linz (Austria); Menden (Germany); Milton Keynes (United Kingdom); Novosibirsk (Russia); Rome (Italy); Schwäbisch Gmünd (Germany); Stoke on Trent (United Kingdom); Södertälje (Sweden); Tournai (Belgium); València (Spain); Wolverhampton (United Kingdom); York (United Kingdom)
Parking management	12	Alcoy (Spain); Badajoz (Spain); Elche (Spain); Heilbronn (Germany); Larissa (Greece); Las Palmas de Gran Canaria (Spain); Linz (Austria); Mons (Belgium); Namur (Namen) (Belgium); Sevilla (Spain); Tallinn (Estonia); Vicenza (Italy)
Improving bus and bike sharing systems	10	Caltanissetta (Italy); Hamburg (Germany); Iasi (Romania); Münster (Germany); Oslo (Norway); Sofia (Bulgaria); Trier (Germany); Valladolid (Spain); Wroclaw (Poland); Zaragoza (Spain)
Traffic control	10	Bocholt (Germany); Bucharest (Romania); Erfurt (Germany); Fuengirola (Spain); Ingolstadt (Germany); Konstanz (Germany); Linz (Austria); Madrid (Spain); Rijeka (Croatia); Zagreb (Croatia)
Autonomous and new mobility systems	8	Bordeaux (France); Hamburg (Germany); Helsinki (Finland); Limerick (Ireland); Pilsen (Czech); Tallinn (Estonia); Toulouse (France); Zaragoza (Spain)
Data integration and digital twins	8	Aachen (Germany); Barcelona (Spain); Jönköping (Sweden); Linz (Austria); Sofia (Bulgaria); Villach (Austria)
Public-facing digital services	7	Artyom (Russia); Jyväskylä (Finland); Lecce (Italy); Linz (Austria); Mytishchi (Russia); Vila-real (Spain); Zagreb (Croatia)

Across these categories, computer vision is one of the most prominent applications of AI. Intelligent cameras are applied to a wide range of operational functions, such as traffic monitoring in Münster, Germany, license-plate recognition in Rimini, Italy and pedestrian counting at intersections in Gdynia and Płock, Poland. Several initiatives use cameras mounted on buses or waste collection vehicles, turning them into mobile sensing platforms for detecting parking violations (Tallinn, Estonia), assessing road and signage conditions (Södertälje, Sweden), and capturing safety-related behaviors such as seatbelt or mobile phone use (Plymouth, United Kingdom). Computer vision is also applied to optimize traffic management, with pilots testing dynamic regulation of traffic lights (Madrid, Spain), detection of congestion zones (Estepona, Spain), and incident identification (Alba Iulia, Romania). In parking management, AI-enabled video systems provide real-time monitoring of space availability (Alcoy, Spain) and surveillance of multi-level structures (Vicenza, Italy). Furthermore, computer vision is applied in the waterborne transport mode, as in The Hague, Netherlands, where cameras classify vessel types and track their movements.

In terms of modes of transport, AI adoptions are concentrated in the road sector ( Figure 2), which accounts for 89% (78 out of 88) of recorded cases. Rail (1 case), air (4 cases), waterborne (2 cases), and cross-modal (3 cases) applications are rare.

## 4. Discussion

By no means does this work represent an exhaustive list of cities adopting AI in urban mobility systems. Rather, it is an effort to fill the gap in our foundational knowledge of where, when, how, and for which modes AI is being adopted. The contribution of this study lies in providing the first systematic and openly accessible documentation of AI adoption in urban mobility across Europe. By combining automated text mining with manual content analysis, this work moves beyond anecdotal accounts and creates a continental-level baseline that policymakers, researchers, and practitioners can use to track and evaluate developments. The database and interactive map not only make the findings transparent and replicable but also establish an open resource that can inform comparative research, support evidence-based policymaking, and help cities learn from one another’s experiences.

The findings highlight both the promise and the fragmentation of AI adoption in urban mobility in Europe. The distribution of deployments shows that AI is not limited to a handful of well-funded metropolitan areas but has diffused across a diverse set of cities in terms of size and location. The temporal dynamics suggest that AI adoption in mobility is still at an early but accelerating stage. The surge in recorded deployments after 2022 coincides with the maturation of data infrastructures and increasing visibility of AI. The adoption is no longer confined to isolated pilots but is beginning to take shape as a recognizable trend. The identified use cases indicate that AI is primarily deployed to improve the operational performance of existing transport systems. The largest categories relate to traffic monitoring and management and to infrastructure condition assessment, followed by parking management, traffic control, and improvements to bus and bike sharing systems. These applications focus on optimizing flows, reducing congestion, improving safety, and supporting predictive maintenance. However, whether these deployments will transition into long-term operational systems remains to be seen. Their sustainability depends on the availability of stable funding, the integration of AI into broader transport strategies, and the development of governance frameworks capable of addressing issues such as accountability, data protection, and public trust.

This work has some limitations. First, the reliance on publicly available official websites means that cases of AI adoption not publicized through these channels may have been overlooked. Second, language differences, even though partially addressed through translation, may have limited the comprehensiveness of the search. Third, the manual content analysis introduces the possibility of coder bias, as judgments about relevance and classification may be influenced by subjective interpretation.

Future work should build on this foundation in several directions. First, the database can be expanded geographically and enriched with additional data sources such as project reports, procurement records, and stakeholder interviews, which would capture cases not visible online and provide richer contextual detail. Second, comparative analyses across different use cases could uncover structural drivers of adoption and common enabling conditions or barriers. Third, longitudinal research is needed to assess whether pilots transition into lasting operations. Methodologically, future studies would benefit from mixed approaches that combine large-scale quantitative tracking of deployments with qualitative insights from practitioners, users, and policymakers. Such integration would allow not only a measurement of the scale and pace of adoption but also a deeper understanding of governance and decision-making processes. Finally, future research should move beyond documenting deployments toward evaluating their broader societal, environmental, and economic impacts, so that AI contributes not only to improving efficiency but also to the development of mobility systems that are sustainable, equitable, and inclusive.

## 5. Conclusions

This study provides the first systematic documentation of AI adoption in urban mobility across Europe. By combining automated text mining with manual content analysis, it maps the geography, timeline, use cases, and transport modes in AI integration. As of September 2025, this work documents a total of 88 AI deployments in urban mobility across 73 cities in 21 European countries. The findings show that AI adoption has accelerated since 2022, is geographically dispersed across Europe, and is concentrated in road-based applications focused on traffic monitoring, infrastructure assessment, and parking management. Overall, deployments tend to support operational control and maintenance of existing infrastructure rather than in transformative reconfigurations of mobility systems. This project enables policymakers, planners, and researchers to monitor developments, compare across cities and modes, and identify emerging trends. More broadly, it contributes to narrowing the gap between theoretical discussions of AI potential and the actual record of real-world deployments. As AI technologies continue to penetrate transport operations and governance structures, this work lays the groundwork for more transparent and evidence-based approaches to shaping the future of AI-mediated urban mobility in Europe.

## Ethics and consent

Ethical approval and consent were not required.

## Software availability statement

Source code available from: https://github.com/caimeng2/UrbMobAI

Archived code available from: https://doi.org/10.5281/zenodo.18712336

License: GNU General Public License.

## Data Availability

Data are available at Zenodo: caimeng2/UrbMobAI: v1.2: https://doi.org/10.5281/zenodo.18712336 ( Cai, 2026). Data are available under the terms of the Creative Commons Attribution 4.0 International license (CC-BY 4.0).
