# Mapping the adoption of artificial intelligence in urban mobility

**DOI:** 10.12688/openreseurope.23222.2

**Published:** 2026-08-24

**Authors:** Meng Cai

**Affiliations:** 1 Department of Civil and Environmental Engineering, TU Darmstadt, Darmstadt, 64287, Germany

**Keywords:** artificial intelligence, AI adoption, AI deployment, urban mobility, transportation, content analysis, Europe, empirical study

## Abstract

Urban transportation systems are undergoing a critical transformation driven by advances in artificial intelligence (AI). Despite many technical and theoretical discussions of AI technologies and a few case studies of AI implementation in selected cities, there remains a large gap in our knowledge about the landscape of the adoption of AI in urban mobility. This study addresses this gap by asking: where, when, how, and for which modes is AI being integrated into urban mobility across Europe? By systematically searching authoritative and publicly available data sources, i.e., the EU Urban Mobility Observatory and official city websites, complemented by manual content analysis, this work documents the geographic distribution, temporal trends, use cases, and transportation modes of AI adoption. As of the end of 2025, the analysis identified 107 AI deployments across 82 cities in 22 European countries. It shows a clear acceleration in adoption after 2022. The deployments fall into eight broad categories: infrastructure condition monitoring, traffic monitoring and analytics, vehicle automation, traffic signal control, public-facing digital services, parking management, transport service operations, and violation detection. In terms of transport mode, adoption is road-centric, with road applications accounting for 89% of recorded cases, while rail, air, waterborne, and cross-modal deployments are relatively rare. The compiled data are openly available online. This work is among the first efforts to establish an empirical base for assessing the prevalence of AI in urban mobility. It aims to provide urban planners, policymakers, and researchers with a better understanding of current AI adoption practices in the European context.

## 1. Introduction

Urban mobility in Europe stands on the cusp of a transformative era as artificial intelligence (AI) and data infrastructures are reshaping transport services ( European Commission, 2025a; Jevinger et al., 2024). A review of academic literature indicates that transportation and mobility are among the sectors most significantly influenced by AI ( Herath & Mittal, 2022). The confluence of large-scale data availability, pressing urban challenges, and policy agendas has paved the way for AI applications, from traffic control optimization, shared autonomous vehicles, to computer-vision enabled road-user behavior detection ( Dilek & Dener, 2023; Jain & Pandey, 2025; Karolemeas et al., 2024; Ventura et al., 2025).

Yet despite a growing number of AI initiatives, the empirical landscape of AI adoption in urban mobility remains under-documented. Existing studies on smart cities and mobility often treat AI as one component of broader digital transformation and focus on its technical potential. The literature tends to describe what AI could do rather than what it actually does in real-world deployment. Recent research shows how AI can enhance routing, traffic flow prediction, and urban planning optimization by taking advantage of large mobility datasets ( Noaeen et al., 2022; Rocco di Torrepadula et al., 2024; Shaygan et al., 2022). The disconnect from real-world deployment is acknowledged within the technical literature itself. In a systematic review of reinforcement learning for network-scale traffic signal control, Noaeen et al. (2022) point out that many studies do not deal with or include real-world data, which challenges the validity of their results. Research has also evaluated AI usage in road vehicle automation and smart traffic control, and noted challenges of trust, explainability, governance capacity, and data availability in real-world deployments ( Englund et al., 2021).

The few empirically grounded studies are often narrow in scope and rarely examine deployments beyond pilot sites. Large-scale empirical analyses across multiple cities and transportation modes are still rare. Two recent studies illustrate the scale at which such work is typically conducted. A case study of five cities on AI usage in Intelligent Transportation Systems found that AI can enhance traffic efficiency, reduce accidents, and support sustainable urban growth, but challenges around data quality, real-time processing, security, public acceptance, and privacy still need to be resolved ( Zemmouchi-Ghomari, 2025). An evaluation of 12 shared automated vehicle pilots in Europe found that shared robotaxis appear safe in closed environments but have more incidents in open, mixed-traffic environment ( Debbaghi et al., 2025). Both studies are informative, but they cover a small number of sites, and neither establishes a baseline against which adoption can be assessed. Debbaghi et al. (2025) themselves call for standardized data collection processes and emphasize the need for baseline data for future deployments.

Taken together, these strands of research point to a critical gap: despite technical potential and proliferating pilots, the record of AI adoption in urban mobility remains fragmented and episodic. This work responds to this gap by constructing an open database of AI adoption in urban mobility systems across European cities. An adoption is counted here when an AI system is operating in an actual urban mobility setting, either as a pilot in the field or as a fully operational service. This work documents four dimensions of AI adoption in Europe: 1) where AI has been deployed; 2) when deployments occurred; 3) how AI is integrated (i.e., application use cases); and 4) which transportation modes are involved (road, rail, air, waterborne, or cross modal).

In doing so, this project bridges the theoretical discussions, technical analyses, and the empirical grounding of AI adoption in urban contexts. The database provides an open resource that enables transport practitioners, researchers, and policymakers to monitor AI deployments, conduct comparative evaluations across place and time, and inform future modeling of AI’s evolving trends in urban mobility.

## 2. Methodology

I used automated keyword searches and manual content analysis to identify cases of AI adoption across Europe. Europe is defined here according to the United Nations M49 classification ( United Nations Statistics Division, n.d.). More specifically, I first used the Google Custom Search Application Programming Interface (API) to locate webpages on the EU Urban Mobility Observatory ( European Commission, 2025b) and on the official websites of 1,462 European cities that mention any of the keywords “artificial intelligence”, “machine learning”, “deep learning”, “neural network”, or “large language model”. These sources were chosen because they are both authoritative and openly available. The list of European city websites was adopted from Karam et al. (2025). To account for language differences, each keyword was translated into the language of each website. Searches were restricted to webpages published on or before December 31, 2025, using the date-range sort parameter of the API. In this step of data collection, I recorded the search date, title, short description, and link of each returned webpage. The short description is the snippet field returned by the API, which is automatically created from the page content and previews the part of the page that best relates to the search query ( Google, 2026). The search code, translations of keywords, and the city websites searched can be found in this project’s repository ( Cai, 2026). The Urban Mobility Observatory search returned 29 webpages, while the search across city websites returned a total of 15,975 webpages mentioning AI keywords.

Next, the title and short description of each webpage were manually reviewed to filter out webpages not related to transportation or mobility. When a webpage was deemed relevant, or when it was unclear whether it was, I examined the full page using the recorded link to determine whether it was truly about real-world AI deployment in urban mobility systems. Feasibility studies, plans, or discussions of potential AI implementation were not counted, as they describe intention rather than practice. When a webpage was considered relevant to AI adoption in urban mobility, the year of adoption, use case, deploying city, and transportation mode were recorded. The recorded use cases were subsequently grouped inductively into broader categories based on the functional similarity of the applications. When the year of adoption was not explicitly stated on a webpage, the latest possible date, for example, the date when the news was published, was used as a proxy.

Finally, the identified AI adoption cases were converted to JSON format and presented as an interactive online map ( https://caimeng2.github.io/UrbMobAI/). Each city with a recorded AI adoption appears as a circular marker sized by case count. Filters allow users to explore adoptions by transportation mode, use case category, and year. Clicking on a marker reveals every case recorded in that city, including the use case, transportation mode, year of adoption, and the source, i.e., the link to the original webpage. In the spirit of open science, the online map and the dataset are released under a Creative Commons Attribution 4.0 International License. Users may freely download the dataset for their own projects.

The methodology of this work has some limitations. First, the source selection bounds what can be observed. The EU Urban Mobility Observatory and official city websites were selected because they are authoritative and openly available, but they capture only what cities choose to publicize. Cities with less developed web communication are likely to be under-represented. Second, although the search covered several AI terms, the selected keywords could not be fully exhaustive. Deployments described only through task-level language such as “smart cameras” without reference to AI may not have been captured. Third, language differences, even though partially addressed through translation, may have limited the comprehensiveness of the search. Fourth, pages judged unrelated to transport or mobility on the basis of their title and description were not opened, which carries the risk that a relevant page with an uninformative title or description was discarded. Fifth, the manual content analysis introduces the possibility of coder bias, as judgments about relevance and classification may be influenced by subjective interpretation.

## 3. Results

**Figure 1.**
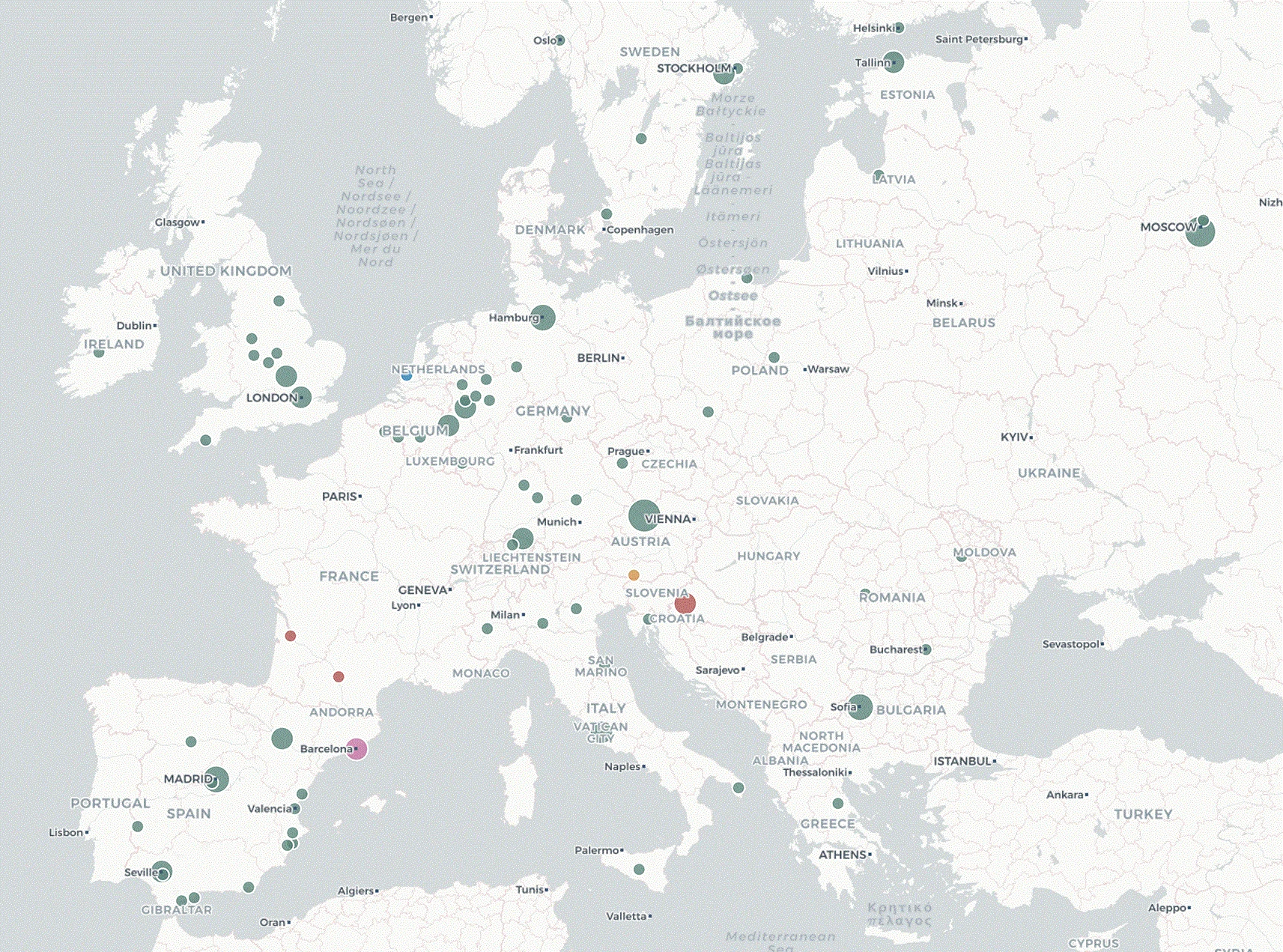
Screenshot of the interactive online map displaying AI adoption in Europe.

As of the end of 2025, this work documents a total of 107 AI deployments in urban mobility across 82 cities in 22 European countries ( Figure 1). The largest numbers of cases are found in Spain (22 cases), Germany (20 cases), and the United Kingdom (10 cases). Deployments are also recorded in Italy (8 cases), Russia (7 cases), Austria (6 cases), Sweden (5 cases), Belgium (3 cases), Bulgaria (3 cases), Croatia (3 cases), Poland (3 cases), Romania (3 cases), Estonia (2 cases), Finland (2 cases), France (2 cases), Norway (2 cases), and in single cases in Czechia, Greece, Ireland, Latvia, the Netherlands, and Switzerland.

**Figure 2.**
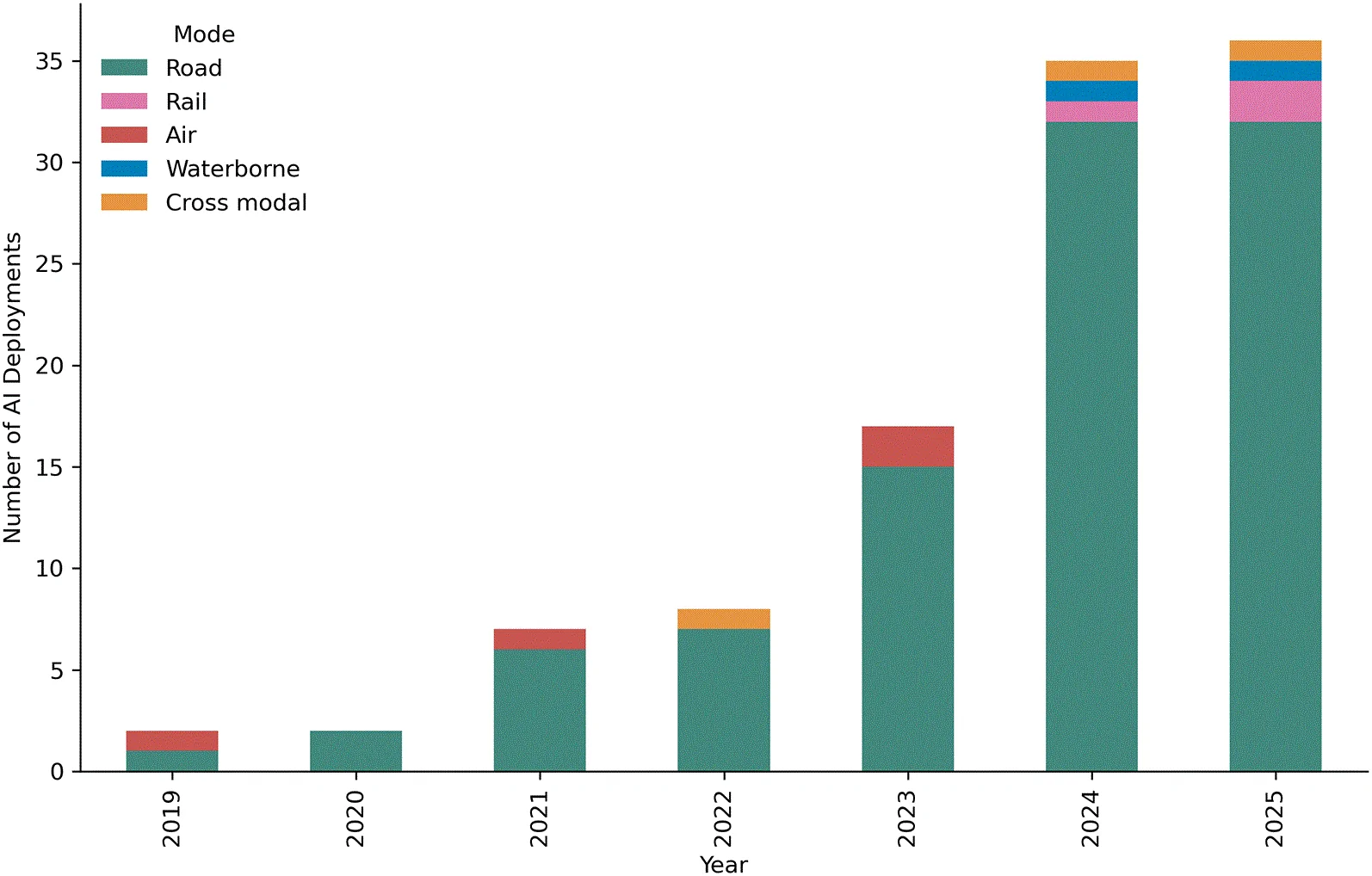
AI deployments in urban mobility by transportation mode over time.

The earliest deployments captured in the database date to 2019 ( Figure 2). Identified AI deployments remained in the single digits between 2019 and 2022 (2 cases in 2019, 2 in 2020, 7 in 2021, and 8 in 2022). Adoption then accelerated sharply, more than doubling to 17 cases in 2023 and again to 35 cases in 2024. A similar level was recorded in 2025 (36 cases).

The AI use cases fall into eight broad categories and cluster primarily around monitoring ( Table 1). Infrastructure condition monitoring (n = 24) and traffic monitoring and analytics (n = 23) are the most prevalent, together accounting for 44% of all cases, followed by vehicle automation (n = 14) and traffic signal control (n = 13). Public-facing digital services (n = 10), parking management (n = 9), transport service operations (n = 8), and violation detection (n = 6) appear less frequently.

**Table 1.** AI use cases identified in European cities.

AI use cases	Count of use cases	City of deployment
Infrastructure condition monitoring	24	Aachen (DE), Duisburg (DE), Düsseldorf (DE), Herne (DE), Konstanz (DE), Leicester (GB), Linz (AT), London (GB), Menden (DE), Milton Keynes (GB), Moscow (RU), Nacka (SE), Novosibirsk (RU), Roma (IT), Schwäbisch Gmünd (DE), Stoke-on-Trent (GB), Södertälje (SE), Tournai (BE), València (ES), Wolverhampton (GB), York (GB)
Traffic monitoring and analytics	23	Aachen (DE), Alba Iulia (RO), Alicante (ES), Almería (ES), Barcelona (ES), Bodø (NO), Coventry (GB), Dos Hermanas (ES), Estepona (ES), Gdynia (PL), Hamburg (DE), Jönköping (SE), London (GB), Madrid (ES), Minden (DE), Móstoles (ES), Płock (PL), Sevilla (ES), Sofia (BG), The Hague (NL), Villach (AT), Winterthur (CH)
Vehicle automation	14	Bordeaux (FR), Hamburg (DE), Helsingborg (SE), Iași (RO), Limerick (IE), Moscow (RU), Plzeň (CZ), Riga (LV), Tallinn (EE), Torino (IT), Toulouse (FR), Wrocław (PL), Zaragoza (ES)
Traffic signal control	13	Bocholt (DE), Bucharest (RO), Fuengirola (ES), Ingolstadt (DE), Konstanz (DE), Linz (AT), Madrid (ES), Milton Keynes (GB), Münster (DE), Rijeka (HR), Roma (IT), Zagreb (HR)
Public-facing digital services	10	Artyom (RU), Barcelona (ES), Düsseldorf (DE), Erfurt (DE), Helsinki (FI), Jyväskylä (FI), Lecce (IT), Mytishchi (RU), Vila-real (ES), Zagreb (HR)
Parking management	9	Alcoy (ES), Badajoz (ES), Heilbronn (DE), Larissa (GR), Las Palmas de Gran Canaria (ES), Linz (AT), Namur (BE), Sevilla (ES), Vicenza (IT)
Transport service operations	8	Caltanissetta (IT), Hamburg (DE), Oslo (NO), Sofia (BG), Södertälje (SE), Trier (DE), Valladolid (ES), Zaragoza (ES)
Violation detection	6	Cremona (IT), Elche (ES), Mons (BE), Plymouth (GB), Rimini (IT), Tallinn (EE)

Across these categories, computer vision is one of the most prominent applications of AI as it underlies much of the monitoring activity. Intelligent cameras are applied to a wide range of operational functions, such as vehicle counting and classification in Winterthur, Switzerland, license-plate recognition in Rimini, Italy, and pedestrian or cyclist counting in Gdynia and Płock, Poland. Several initiatives use cameras mounted on buses or waste collection vehicles, turning them into mobile sensing platforms for detecting parking violations (Tallinn, Estonia) and assessing road and signage conditions (Södertälje, Sweden). Roadside systems support enforcement, capturing safety-related behaviors such as failure to wear a seatbelt or mobile phone use (Plymouth, United Kingdom). Computer vision is also applied to optimize traffic management, with pilots testing dynamic regulation of traffic lights (Madrid, Spain) and detection of high-density traffic zones (Estepona, Spain). In parking management, AI-enabled video systems provide real-time monitoring of space availability (Alcoy, Spain) and surveillance of a multi-level car park (Vicenza, Italy). Furthermore, computer vision is applied in the waterborne transport mode, as in The Hague, Netherlands, where cameras classify vessel types and assess their course and speed.

In terms of modes of transport, AI adoptions are concentrated in the road sector ( Figure 2), which accounts for 89% (95 out of 107) of recorded cases. Air (4 cases), rail (3 cases), cross-modal (3 cases), and waterborne (2 cases) applications are rare.

## 4. Discussion

By no means does this work represent an exhaustive list of cities adopting AI in urban mobility systems. Rather, it is an effort to fill the gap in our foundational knowledge of where, when, how, and for which modes AI is being adopted. The contribution of this study lies in providing the first systematic and openly accessible documentation of AI adoption in urban mobility across Europe. By combining automated keyword searches with manual content analysis, this work moves beyond anecdotal accounts and creates a continental-level baseline that policymakers, researchers, and practitioners can use to track and evaluate developments. The database and interactive map not only make the findings transparent and replicable but also establish an open resource that can inform comparative research, support evidence-based policymaking, and help cities learn from one another’s experiences.

The findings highlight both the promise and the fragmentation of AI adoption in urban mobility in Europe. The distribution of deployments shows that AI is not limited to a handful of well-funded metropolitan areas but has diffused across a diverse set of cities in terms of size and location. This suggests that AI adoption is not exclusively determined by metropolitan scale or by the resources typically associated with large cities, though the specific capabilities that allow smaller municipalities to adopt AI systems fall outside the scope of the present work. The temporal dynamics suggest that AI adoption in mobility is still at an early but accelerating stage. The surge in recorded deployments after 2022 coincides with the maturation of data infrastructures and increasing visibility of AI. Adoption is no longer confined to isolated pilots but is beginning to take shape as a recognizable trend.

The identified use cases indicate that AI is primarily deployed to improve the performance of existing transport systems. The largest categories relate to infrastructure condition monitoring and to traffic monitoring and analytics, followed by vehicle automation and traffic signal control. These applications focus on observing existing assets and flows, supporting predictive maintenance, reducing congestion, and improving safety. However, whether these deployments will transition into long-term operational systems remains to be seen. Their sustainability depends on the availability of stable funding, the integration of AI into broader transport strategies, and the development of governance frameworks capable of addressing issues such as accountability, data protection, and public trust.

Road-based deployments account for the majority of all recorded cases, while rail, air, waterborne, and cross-modal applications are relatively rare. This pattern is consistent with, and helps explain, the use-case findings reported above: infrastructure condition monitoring and traffic monitoring and analytics, the two largest categories, both rely on computer-vision and sensor technologies that are comparatively easy to retrofit onto existing road assets. The same road-centric emphasis is consistent with existing empirical studies. Debbaghi et al. (2025) and Zemmouchi-Ghomari (2025) both build their empirical accounts of AI-enabled mobility around road-based systems. Part of the imbalance may also be due to this work’s methodology: cities typically own and operate road infrastructure directly, whereas rail, air, and waterborne services are more often run by national or regional operators whose AI initiatives may not be documented on the municipal websites searched here, a point already noted as a limitation of the source selection.

The European Union’s policy landscape offers additional context for both the timing and the concentration of AI deployments. The Digital Decade Policy Programme 2030 ( Decision (EU) 2022/2481, 2022) established common objectives for digital infrastructure and public services. The Sustainable and Smart Mobility Strategy ( COM(2020) 789 Final, 2020) and the subsequent New EU Urban Mobility Framework ( COM(2021) 811 Final, 2021) explicitly promote the deployment of intelligent transport systems and smart urban mobility solutions. The 2023 revision of the Intelligent Transport Systems Directive ( Directive (EU) 2023/2661, 2023) specifically calls for faster deployment of intelligent, data-driven road services. The AI Act ( Regulation (EU) 2024/1689, 2024), which entered into force in 2024, added a harmonized and risk-based governance framework. These policies may have increased institutional attention, data readiness, and regulatory clarity, thereby creating more favorable conditions for the adoption and reporting of AI applications. However, they should be interpreted as an enabling context rather than as a direct cause of the rise in AI adoption.

Future work should build on this foundation in several directions. First, the database can be expanded geographically and enriched with additional data sources such as project reports, procurement records, and stakeholder interviews, which would capture cases not visible online and provide richer contextual detail. Second, comparative analyses across different use cases and transportation modes could uncover structural drivers of adoption and common enabling conditions or barriers, such as a city’s socio-economic profile, administrative capacity, public support, and funding access. Third, longitudinal research is needed to assess whether pilots transition into lasting operations. Methodologically, future studies would benefit from mixed methods approaches that combine large-scale quantitative tracking of deployments with qualitative insights from practitioners, users, and policymakers. Such integration would allow not only measurement of the scale and pace of adoption but also a deeper understanding of governance and decision-making processes. Finally, future research should move beyond documenting deployments toward evaluating their broader societal, environmental, and economic impacts, so that AI contributes not only to improving efficiency but also to the development of mobility systems that are sustainable, equitable, and inclusive.

## 5. Conclusions

This study provides the first systematic documentation and openly accessible of AI adoption in urban mobility across Europe. By combining automated keyword searches with manual content analysis, it maps the geography, timeline, use cases, and transport modes in AI integration. As of the end of 2025, this work documents a total of 107 AI deployments in urban mobility across 82 cities in 22 European countries. The findings show that AI adoption has accelerated since 2022, is geographically dispersed across cities of varying size and location and is concentrated in road-based applications focused on infrastructure condition monitoring, traffic monitoring and analytics, and traffic signal control. Overall, deployments tend to support maintenance of existing infrastructure and operational control rather than in transformative reconfiguration of mobility systems. This project enables policymakers, planners, and researchers to monitor developments, compare across cities and modes, and identify emerging trends. More broadly, it contributes to narrowing the gap between theoretical discussions of AI potential and the actual record of real-world deployments. As AI technologies continue to penetrate transport operations and governance structures, this work lays the groundwork for more transparent and evidence-based approaches to shaping the future of AI-mediated urban mobility in Europe.

## Ethics and consent

Ethical approval and consent were not required.

## Software availability statement

Source code available from: https://github.com/caimeng2/UrbMobAI

Archived code available from: https://doi.org/10.5281/zenodo.18711974

License: GNU General Public License.

## Data Availability

Data are available at Zenodo: caimeng2/UrbMobAI: v1.2: https://doi.org/10.5281/zenodo.18712336 ( Cai, 2026). Data are available under the terms of the Creative Commons Attribution 4.0 International license (CC-BY 4.0).
