# Supplementary material for: Mapping the adoption of artificial intelligence in urban mobility
Source: Open Res Eur. 2026 Aug 24;6:70. Originally published 2026 Mar 10. [Version 2] doi: 10.12688/openreseurope.23222.2 (PMC13562952; doi:10.12688/openreseurope.23222.2)
Supplement: Peer review history

# Reviewer report: Reviewer response for version 2

**DOI:** 10.21956/openreseurope.27034.r79620

**Published:** 2026-09-08

**Authors:** Alan Miguel Valdez

**Affiliations:** 1 The Open University, Milton Keynes, UK

Having read version 2 of this research article, I am happy to recommend its indexing. I read the original publication with interest and I consider that version 2 has been much improved by the addition of various keywords associated with the use of urban AI in transport and mobility, as well as by the consideration of additional data for 2025 which now allows for like-for-like comparison between the years covered by the study. This article makes a valuable contribution by providing a provide a concise, timely survey of the use of AI to support urban mobility in Europe. This work is grounded on an appropriate overview of academic and policy literature, and the provision of source code as well as data produced with it support replicability. As the author has observed, there is much discussion of what AI could do but not enough of what it is already doing, with this work making a valuable contribution towards addressing this gap in knowledge.

Is the study design appropriate and does the work have academic merit?

Partly

Is the work clearly and accurately presented and does it cite the current literature?

Yes

If applicable, is the statistical analysis and its interpretation appropriate?

Not applicable

Are all the source data underlying the results available to ensure full reproducibility?

Yes

Are the conclusions drawn adequately supported by the results?

Partly

Are sufficient details of methods and analysis provided to allow replication by others?

Yes

Reviewer Expertise:

NA

I confirm that I have read this submission and believe that I have an appropriate level of expertise to confirm that it is of an acceptable scientific standard.

# Reviewer report: Reviewer response for version 2

**DOI:** 10.21956/openreseurope.27034.r79619

**Published:** 2026-09-08

**Authors:** Sila Ceren Varis Husar

**Affiliations:** 1 Slovak University of Technology in Bratislava, Bratislava, Slovakia

I have no further comments to make, I would like to thank author to take the previous comments seriously and to make necessary changes with the article.

Is the study design appropriate and does the work have academic merit?

Yes

Is the work clearly and accurately presented and does it cite the current literature?

Partly

If applicable, is the statistical analysis and its interpretation appropriate?

Partly

Are all the source data underlying the results available to ensure full reproducibility?

Partly

Are the conclusions drawn adequately supported by the results?

Yes

Are sufficient details of methods and analysis provided to allow replication by others?

No

Reviewer Expertise:

Urban planning, regional planning, statistical analysis in planning

I confirm that I have read this submission and believe that I have an appropriate level of expertise to confirm that it is of an acceptable scientific standard.

# Reviewer report: Reviewer response for version 2

**DOI:** 10.21956/openreseurope.27034.r79621

**Published:** 2026-09-08

**Authors:** Liang Zhao

**Affiliations:** 1 Kyoto University, Kyoto, Japan

I don't have further comment. The author has answered all my previous comments in a professional way. Thanks.

Is the study design appropriate and does the work have academic merit?

Yes

Is the work clearly and accurately presented and does it cite the current literature?

Partly

If applicable, is the statistical analysis and its interpretation appropriate?

Yes

Are all the source data underlying the results available to ensure full reproducibility?

Yes

Are the conclusions drawn adequately supported by the results?

Yes

Are sufficient details of methods and analysis provided to allow replication by others?

Partly

Reviewer Expertise:

Machine learning, algorithms, network science

I confirm that I have read this submission and believe that I have an appropriate level of expertise to confirm that it is of an acceptable scientific standard.

# Reviewer report: Reviewer response for version 1

**DOI:** 10.21956/openreseurope.25126.r71031

**Published:** 2026-04-16

**Authors:** Sila Ceren Varis Husar

**Affiliations:** 1 Slovak University of Technology in Bratislava, Bratislava, Slovakia

Article starts with a good overall introduction for the critical gap in the knowledge on AI adoption in urban mobility. The research topic is highly relevant and timely. For a general audience, it would be beneficial to explain shortly how text mining works and how the researcher employed specifically in this research.

- About methodology, AI in different languages could be explained more. How AI interpreted and used in Hungarian language is a good example and if the different usages of AI is explained more in other languages, it will widen the information.
- There are some unclear aspects of the methodology, 13423 webpages have been manually reviewed? Could you please specify more what real-world AI deployment entails in the introduction part? Even if the feasibility plans were not considered as real-world AI deployment, I think there is a potential in some of them if they are concrete steps towards the adoption.
- Please add these dimensions to the limitations via underlining the data selection, filtered data and reasons; and move limitations to the methodology section rather than discussion part. The policy dimension on how the AI transforms the urban mobility is missing only by referring to the EU Urban Mobility Observatory website.
- I would be interested to see more the rules and regulations or even attempts to understand and approach the AI in urban mobility on European level. Deployment eight broad categories – would they change if the dataset changes? Referring to table 1 Count of AI use cases, are there any more groups with counts of less than 7?
- Descriptive part of the analysis has more potential to tell us the general overview of the AI adoption. For example the highest concentration of adoption is 89% in road sector is a good take away for discussion. Would it be possible to extend the findings in this direction?
- The paper takes a good snapshot of the current situation of AI adoption, which I believe many work can use as a reference and build upon it. I find this finding very important “The distribution of deployments shows that AI is not limited to a handful of well-funded metropolitan areas but has diffused across a diverse set of cities in terms of size and location.” This could supports and opens up more discussions about comparisons of sizes and capabilities of places in AI adoption.
- The main strength of this research for me is that it is openly accessible and being reviewed. I would suggest sharing on more platforms and collecting feedback of the mentioned case studies and even revealing and adding undetected cases that have AI adoption in urban mobility. End of discussion part, future research possibilities are listed out extensively but I would suggest to keep in track of the first vein where extension of database and initiating synergies with different cases across Europe.

Is the study design appropriate and does the work have academic merit?

Yes

Is the work clearly and accurately presented and does it cite the current literature?

Partly

If applicable, is the statistical analysis and its interpretation appropriate?

Partly

Are all the source data underlying the results available to ensure full reproducibility?

Partly

Are the conclusions drawn adequately supported by the results?

Yes

Are sufficient details of methods and analysis provided to allow replication by others?

No

Reviewer Expertise:

Urban planning, regional planning, statistical analysis in planning

I confirm that I have read this submission and believe that I have an appropriate level of expertise to confirm that it is of an acceptable scientific standard, however I have significant reservations, as outlined above.

# Response

**Published:** 2026-08-14

**Authors:** Meng Cai

**Affiliations:** Technische Universitat Darmstadt, Darmstadt, Hesse, Germany

**Article starts with a good overall introduction for the critical gap in the knowledge on AI adoption in urban mobility. The research topic is highly relevant and timely. For a general audience, it would be beneficial to explain shortly how text mining works and how the researcher employed specifically in this research.**

 Hi Dr Varis Husar, Thank you for your kind words and for flagging the term text mining. The methodology should not have been described as text mining, and I have corrected the term throughout the article and now describe the approach as automated keyword searches combined with manual content analysis. Text mining means the computational extraction of information or patterns from text, using automated classification, topic modelling, etc. This study does not do that. I use the Google Custom Search API to retrieve webpages matching a set of keywords, record the title, the description, and the link, and then review and code them by hand. The automation lies in the retrieval, not in the analysis of the text. Automated classification was my initial plan, but I dropped it later because the volume of data was manageable for manual review. Most of the returned results are obviously unrelated to transport and can be excluded from the title and description alone. Manual coding is better in my opinion, since every judgement is traceable to a source link.

**•  About methodology, AI in different languages could be explained more. How AI interpreted and used in Hungarian language is a good example and if the different usages of AI is explained more in other languages, it will widen the information.**

 Thanks for pointing this out. I used Hungarian as an example only because this study was first presented at the Transport Research Arena in Budapest. In the revised manuscript, I have added the full list of keywords searched on each website to this study's repository here: https://github.com/caimeng2/UrbMobAI/blob/master/search/euro_cities.csv

**• There are some unclear aspects of the methodology, 13423 webpages have been manually reviewed? Could you please specify more what real-world AI deployment entails in the introduction part? Even if the feasibility plans were not considered as real-world AI deployment, I think there is a potential in some of them if they are concrete steps towards the adoption.**

 Thank you for these questions! Regarding the manual review, not all webpages were reviewed in full. I first checked the short descriptions and titles to see whether a webpage was about transport. The short description is generated by Google from the page content and previews the part of the page that best relates to the search query. I opened the link and reviewed the webpage itself only when the page was about transportation, or when I could not tell from the title or the description whether the content was about transport. I did not count feasibility plans because they describe intentions and may or may not lead to an actual deployment. I have clarified the process in the revised manuscript: the title and short description of each webpage were manually reviewed to filter out webpages not related to transportation or mobility. When a webpage was deemed relevant, or when it was unclear whether it was, I examined the full page using the recorded link to determine whether it was truly about real-world AI deployment in urban mobility systems. Feasibility studies, plans, or discussions of potential AI implementation were not counted, as they describe intention rather than practice. And yes, I have added what real-world AI deployment entails in the introduction in the revised manuscript: This work responds to this gap by constructing an open database of AI adoption in urban mobility systems across European cities. An adoption is counted here when an AI system is operating in an actual urban mobility setting, either as a pilot in the field or as a fully operational service.

**• Please add these dimensions to the limitations via underlining the data selection, filtered data and reasons; and move limitations to the methodology section rather than discussion part. The policy dimension on how the AI transforms the urban mobility is missing only by referring to the EU Urban Mobility Observatory website.**

 Thank you for these suggestions, all three of which I have adopted. On the limitations of data selection and filtering. I have expanded the limitations to state explicitly which sources were selected and why, and what was filtered out: First, the source selection bounds what can be observed. The EU Urban Mobility Observatory and official city websites were selected because they are authoritative and openly available, but they capture only what cities choose to publicize. Cities with less developed web communication are likely to be under-represented. Second, although the search covered several AI terms, the selected keywords could not be fully exhaustive. Deployments described only through task-level language such as “smart cameras” without reference to AI may not have been captured. Third, ... Fourth, pages judged unrelated to transport or mobility on the basis of their title and description were not opened, which carries the risk that a relevant page with an uninformative title or description was discarded. Fifth, … On the placement of the limitations. I agree that the limitations belong in the Methodology. They now sit at the end of Section 2 and have been removed from the Discussion. On the policy dimension. I have added a paragraph in the discussion: The European Union’s policy landscape offers additional context for both the timing and the concentration of AI deployments. The Digital Decade Policy Programme 2030 (Decision (EU) 2022/2481, 2022) established common objectives for digital infrastructure and public services. The Sustainable and Smart Mobility Strategy (COM(2020) 789 Final, 2020) and the subsequent New EU Urban Mobility Framework (COM(2021) 811 Final, 2021) explicitly promote the deployment of intelligent transport systems and smart urban mobility solutions. The 2023 revision of the Intelligent Transport Systems Directive (Directive (EU) 2023/2661, 2023) specifically calls for faster deployment of intelligent, data-driven road services. The AI Act (Regulation (EU) 2024/1689, 2024), which entered into force in 2024, added a harmonized and risk-based governance framework. These policies may have increased institutional attention, data readiness, and regulatory clarity, thereby creating more favorable conditions for the adoption and reporting of AI applications. However, they should be interpreted as an enabling context rather than as a direct cause of the rise in AI adoption.

**• I would be interested to see more the rules and regulations or even attempts to understand and approach the AI in urban mobility on European level. Deployment eight broad categories – would they change if the dataset changes? Referring to table 1 Count of AI use cases, are there any more groups with counts of less than 7?**

 Thank you for this question. The eight categories in Table 1 were derived inductively from the individual use cases, grouped by functional similarity. So you are right, the categories are a product of the current dataset and could evolve. In this revision, I also refined the categories with the additional data, which changed several category counts. Following this revision, only one category, violation detection (n = 6), falls below a count of seven; all the categories are listed in Table 1 with their full counts.

**• Descriptive part of the analysis has more potential to tell us the general overview of the AI adoption. For example the highest concentration of adoption is 89% in road sector is a good take away for discussion. Would it be possible to extend the findings in this direction?**

 Thank you for this great suggestion. I have added a new paragraph to discuss this: Road-based deployments are the majority of all recorded cases, while rail, air, waterborne, and cross-modal applications are relatively rare. This pattern is consistent with, and helps explain, the use-case findings reported above: infrastructure condition monitoring and traffic monitoring and analytics, the two largest categories, both rely on computer-vision and sensor technologies that are comparatively easy to retrofit onto existing road assets. The same road-centric emphasis is consistent with existing empirical studies. Debbaghi et al. (2025) and Zemmouchi-Ghomari (2025) both build their empirical accounts of AI-enabled mobility around road-based systems. Part of the imbalance may also be due to this work's methodology: cities usually directly own and operate road infrastructure, whereas rail, air, and waterborne services are more often run by national or regional operators whose AI initiatives may not be documented on the municipal websites searched here, a point already noted as a limitation of the source selection.

**• The paper takes a good snapshot of the current situation of AI adoption, which I believe many work can use as a reference and build upon it. I find this finding very important “The distribution of deployments shows that AI is not limited to a handful of well-funded metropolitan areas but has diffused across a diverse set of cities in terms of size and location.” This could supports and opens up more discussions about comparisons of sizes and capabilities of places in AI adoption.**

 Thank you for this positive feedback! I have expanded the Discussion section to clarify that the observed distribution suggests that AI adoption is not exclusively determined by metropolitan scale or by the resources typically associated with large cities. At the same time, I acknowledge that the present study does not assess the specific capabilities that enable smaller municipalities to adopt AI systems and therefore cannot determine whether cities of different sizes adopt AI under comparable conditions. In addition, I have expanded the future research discussion to call for comparative analyses of the structural drivers and barriers affecting adoption across cities, use cases, and transportation modes.

**• The main strength of this research for me is that it is openly accessible and being reviewed. I would suggest sharing on more platforms and collecting feedback of the mentioned case studies and even revealing and adding undetected cases that have AI adoption in urban mobility. End of discussion part, future research possibilities are listed out extensively but I would suggest to keep in track of the first vein where extension of database and initiating synergies with different cases across Europe.**

 Thank you for taking the time to review this paper and for all the constructive feedback! I hope you find the revised manuscript greatly improved. I will look for more opportunities and platforms to share this work and the database/map. My goal is to update this regularly as time and capacity allow. Ultimately, I envision this project as a living archive of AI adoption in urban mobility, a resource that can grow over time to track new deployments, shifting use cases, and broader trends.

# Reviewer report: Reviewer response for version 1

**DOI:** 10.21956/openreseurope.25126.r71022

**Published:** 2026-04-07

**Authors:** Liang Zhao

**Affiliations:** 1 Kyoto University, Kyoto, Japan

I found this study and its output are valuable. It maps the adoption of AI in urban mobility across Europe. Utilizing a hybrid method of Google Custom Search API text mining and manual content analysis, the author surveyed 1,448 city websites and the EU Urban Mobility Observatory. The study identified 88 AI deployments in 73 cities across 21 countries as of September 2025. Key findings indicate a sharp acceleration in adoption starting in 2022, a heavy concentration on road-based applications (89%), and primary use cases centered on traffic management and infrastructure condition assessment.

 On the other hand, I found some details are not clear or not enough. I list them in the following for author's reference:

 1. Describe the detail how the "short description" for each webpage returned by Google Custom Search was generated.

 2. Better to provide a supplementary appendix containing the full list of 1,448 city websites and the exact translation of search keywords used for each language.

 3. (Optional) Clarify the API settings and how the search was throttled to comply with Google’s usage limits.

 4. (Optional) Replace Figure 1 with a high-resolution, static geographic map (e.g., generated via GIS) that displays city clusters or adoption density, rather than web-app (?) screenshot.

 5. Add a discussion on the role of the EU AI Act (2024) and the Digital Decade policy agenda in driving the observed peak in 2024 deployments. This would provide a necessary policy context for the quantitative trends observed.

 6. (Same as a comment provided by the other reviewer) There can be many keywords in the AI field other than "artificial intelligence." Suggest to research with more keywords such as "machine learning," "computer vision," "image processing," etc. For following works (not this one), I suggest the author also include "large language model" (LLM) as well.

 7. Related to the above, the author concluded that "computer vision" is one of the most prominent application of AI but did not list what others are. A reader may wonder if other keywords were considered at all.

 By addressing these transparency and contextual gaps, the paper will provide a robust and replicable foundation for future research in urban AI adoption.

Is the study design appropriate and does the work have academic merit?

Yes

Is the work clearly and accurately presented and does it cite the current literature?

Partly

If applicable, is the statistical analysis and its interpretation appropriate?

Yes

Are all the source data underlying the results available to ensure full reproducibility?

Yes

Are the conclusions drawn adequately supported by the results?

Yes

Are sufficient details of methods and analysis provided to allow replication by others?

Partly

Reviewer Expertise:

Machine learning, algorithms, network science

I confirm that I have read this submission and believe that I have an appropriate level of expertise to confirm that it is of an acceptable scientific standard, however I have significant reservations, as outlined above.

# Response

**Published:** 2026-08-14

**Authors:** Meng Cai

**Affiliations:** Technische Universitat Darmstadt, Darmstadt, Hesse, Germany

Hi Dr. Zhao,

 Thank you for taking the time to review this manuscript! I have addressed your comments point by point below.

**On the other hand, I found some details are not clear or not enough. I list them in the following for author's reference:**

**1. Describe the detail how the "short description" for each webpage returned by Google Custom Search was generated.**

 Thanks for pointing this out. I’ve added this detail in the revised methodology section: The short description is the snippet field returned by the API, which is automatically created from the page content and previews the part of the page that best relates to the search query (Google, 2026).

**2. Better to provide a supplementary appendix containing the full list of 1,448 city websites and the exact translation of search keywords used for each language.**

 Done. I’ve added the list of city websites and the keyword translations to this project’s repo: https://github.com/caimeng2/UrbMobAI/blob/master/search/euro_cities.csv I miscalculated the number of city websites in the original version. It should be 1,462, which I have corrected in the revised manuscript.

**3. (Optional) Clarify the API settings and how the search was throttled to comply with Google’s usage limits.**

 Thank you for the suggestion. In this round of revision, I added an end date parameter, which I now describe in the Methodology section: The search was restricted to webpages published on or before December 31, 2025, using the date-range sort parameter of the API. I did not set any other parameters. As for usage limits, the Google Custom Search API allows up to 10,000 queries per day, and this search stayed well below that.

**4. (Optional) Replace Figure 1 with a high-resolution, static geographic map (e.g., generated via GIS) that displays city clusters or adoption density, rather than web-app (?) screenshot.**

 Thank you for the suggestion. I gave this some thought but decided to keep the screenshot. My reason is that the figure is meant to direct readers to the online map, which carries more information than a static map can: each marker links to the use case, transport mode, year, and the original source. In this revision, I have also added filter and search functions, so the map is much easier to explore. You are right, however, that the previous screenshot was low in resolution. I have replaced it with a higher-resolution image in the revised manuscript.

**5. Add a discussion on the role of the EU AI Act (2024) and the Digital Decade policy agenda in driving the observed peak in 2024 deployments. This would provide a necessary policy context for the quantitative trends observed.**

 Thank you for the suggestion. I have added a paragraph in the discussion regarding the policy context: The European Union’s policy landscape offers additional context for both the timing and the concentration of AI deployments. The Digital Decade Policy Programme 2030 (Decision (EU) 2022/2481, 2022) established common objectives for digital infrastructure and public services. The Sustainable and Smart Mobility Strategy (COM(2020) 789 Final, 2020) and the subsequent New EU Urban Mobility Framework (COM(2021) 811 Final, 2021) explicitly promote the deployment of intelligent transport systems and smart urban mobility solutions. The 2023 revision of the Intelligent Transport Systems Directive (Directive (EU) 2023/2661, 2023) specifically calls for faster deployment of intelligent, data-driven road services. The AI Act (Regulation (EU) 2024/1689, 2024), which entered into force in 2024, added a harmonized and risk-based governance framework. These policies may have increased institutional attention, data readiness, and regulatory clarity, thereby creating more favorable conditions for the adoption and reporting of AI applications. However, they should be interpreted as an enabling context rather than as a direct cause of the rise in AI adoption.

**6. (Same as a comment provided by the other reviewer) There can be many keywords in the AI field other than "artificial intelligence." Suggest to research with more keywords such as "machine learning," "computer vision," "image processing," etc. For following works (not this one), I suggest the author also include "large language model" (LLM) as well.**

 Thanks for pointing it out. I totally agree that more keywords should be included. The search now uses "artificial intelligence" OR "machine learning" OR "deep learning" OR "neural network" OR "large language model," each translated per website language. As you suggest, "large language model" is already among them. These terms were chosen because they align with the EU definition of AI, which states that machine learning approaches should be understood as AI techniques, describes deep learning as a subset of machine learning using neural network architectures, and treats language models as AI systems (European Commission, 2025). I did not add "computer vision" or "image processing" because neither necessarily involves AI. Earlier computer vision systems can be based on methods such as background subtraction or edge detection (Premaratne et al., 2023), which are image processing techniques rather than AI.

**7. Related to the above, the author concluded that "computer vision" is one of the most prominent application of AI but did not list what others are. A reader may wonder if other keywords were considered at all.**

 Thank you, this is a fair point. Computer vision was singled out because the two largest categories, infrastructure condition monitoring and traffic monitoring, largely depend on cameras and computer vision. I have made the basis for that claim explicit in the revision: Across these categories, computer vision is one of the most prominent applications of AI as it underlies much of the monitoring activity. On keywords, I didn't search “computer vision.” This is a pattern that emerged from the results.

**By addressing these transparency and contextual gaps, the paper will provide a robust and replicable foundation for future research in urban AI adoption.**

 Thank you! This is exactly what I was hoping the paper could offer. I hope you find the revised version improved.  

 References European Commission. (2025). *Guidelines on the definition of an artificial intelligence system established by Regulation (EU) 20241689 (AI Act)*. https://digital-strategy.ec.europa.eu/en/library/commission-publishes-guidelines-ai-system-definition-facilitate-first-ai-acts-rules-application Google. (2026). *How to Write Meta Descriptions | Google Search Central | Documentation*. Google for Developers. https://developers.google.com/search/docs/appearance/snippet Premaratne, P., Jawad Kadhim, I., Blacklidge, R., & Lee, M. (2023). Comprehensive review on vehicle Detection, classification and counting on highways. *Neurocomputing*, *556*, 126627. https://doi.org/10.1016/j.neucom.2023.126627

# Reviewer report: Reviewer response for version 1

**DOI:** 10.21956/openreseurope.25126.r71027

**Published:** 2026-03-25

**Authors:** Alan Miguel Valdez

**Affiliations:** 1 The Open University, Milton Keynes, UK

I read this article with interest. This contribution begins to provide a concise, timely survey of the use of AI to support urban mobility in Europe. The research is grounded on an appropriate overview of academic and policy literature, and the provision of source code as well as data produced with it support replicability. As the author mentioned, there is much discussion of what AI could do but not enough of what it is already doing. I believe this article will make a valuable contribution to the literature but I have concerns about the specific queries used to reach the results.

 First, I note that the author has used the narrow keyword "Artificial Intelligence". However, there are other, more specialised terms which are functionally identical to AI or refer to more specific applications of the broad label "AI". Examples include "computer vision" and "machine learning". This matters because the author's query as phrased is likely to miss many relevant projects, including for example one for machine learning in Vilnius and one on machine learning in Debrecen which are immediately findable in the observatory. I would strongly suggest for the author to draw on the literature to identify other keywords associated with the use of urban AI in transport and mobility. 

 A second concern is that the criteria used by the author to consider that a city should be considered European is not clear. I was somewhat surprised to see deployments in the UK and Russia included in the results. The author mentioned using a list of cities adapted from Karam et al. Perhaps a clearer explanation on the nature of this adaption would be useful. I note that the spreadsheet provided by Karam et al groups together cities from Japan and Europe, and draws on a broad definition of Europe (again, including for example the UK and Russia). Although I will be very happy about seeing British projects included, I wonder if a focus on the European Union would be more appropriate for this publication. 

 As much of the methodology for this study relies on automation, I hope code can be adapted and queries can be re-run with relative ease, although I realise that the results and conclusions will have to be adjusted. 

 I have some minor concerns in addition to the above, which are very minor but I will mention for the sake of completeness.

 - "I identified 88 AI deployments" should probably be rephrased to avoid "I". Perhaps "The analysis identified"? 

 "It shows a clear acceleration in adoption...". I am not sure what "it" refers to. (But it can refer to the analysis, if the change I suggested is implemented. 

 The point of some of the examples mentioned in the introduction is not clear. Paragraphs 2 and 3, which discuss Noaen et al, Zemmouchi-Ghomari and Debbaghi et al feel like random examples, with no clear sense of why they were included. Are these major projects, or particularly illustrative? 

 Finally, I also that the authors discussion of year to year trends loses credibility because it was completed 9 months into 2025. A revision would provide an excellent opportunity to provide full data for that year, allowing for a more robust analysis of trends.

Is the study design appropriate and does the work have academic merit?

Partly

Is the work clearly and accurately presented and does it cite the current literature?

Yes

If applicable, is the statistical analysis and its interpretation appropriate?

Not applicable

Are all the source data underlying the results available to ensure full reproducibility?

Yes

Are the conclusions drawn adequately supported by the results?

Partly

Are sufficient details of methods and analysis provided to allow replication by others?

Yes

Reviewer Expertise:

Urban AI, Smart and Sustainable Transport, Urban Experiments, Future Urban Environments.

I confirm that I have read this submission and believe that I have an appropriate level of expertise to confirm that it is of an acceptable scientific standard, however I have significant reservations, as outlined above.

# Response

**Published:** 2026-08-14

**Authors:** Meng Cai

**Affiliations:** Technische Universitat Darmstadt, Darmstadt, Hesse, Germany

Hi Dr Miguel Valdez, thank you for your kind words and for taking the time to review this paper. I am glad that you see the value of the project, and I greatly appreciate your constructive feedback. Please see my point-by-point response to your comments below.

*First, I note that the author has used the narrow keyword "Artificial Intelligence". However, there are other, more specialised terms which are functionally identical to AI or refer to more specific applications of the broad label "AI". Examples include "computer vision" and "machine learning". This matters because the author's query as phrased is likely to miss many relevant projects, including for example one for machine learning in Vilnius and one on machine learning in Debrecen which are immediately findable in the observatory. I would strongly suggest for the author to draw on the literature to identify other keywords associated with the use of urban AI in transport and mobility.*

**Response: **Thanks for pointing it out. I totally agree that more keywords should be included. The search now uses "artificial intelligence" OR "machine learning" OR "deep learning" OR "neural network" OR "large language model," each translated per website language. These terms were chosen because they align with the EU definition of AI, which states that machine learning approaches should be understood as AI techniques, describes deep learning as a subset of machine learning using neural network architectures, and treats language models as AI systems (European Commission, 2025). I did not add "computer vision" because it does not necessarily involve AI. Earlier computer vision systems can be based on background subtraction or edge detection (Premaratne et al., 2023). I didn’t see concrete deployed AI use cases in Vilnius or Debrecen, though. I assume the articles you refer to are https://urban-mobility-observatory.transport.ec.europa.eu/building-greener-smarter-transport-system-vilnius_en and https://urban-mobility-observatory.transport.ec.europa.eu/resources/case-studies/case-study-effect-machine-learning-congestion-handling-debrecen-hungary_en.  The article on Vilnius describes several digital and data-driven initiatives but does not explicitly say that Vilnius uses AI or machine learning. The article on Debrecen is more of a case study conducted by a university rather than a deployment by the city. Even with the revised keywords, I'm sure I'm still missing some use cases. I've noted this in the limitations: Second, although the search covered several AI terms, the selected keywords could not be fully exhaustive. Deployments described only through task-level language such as “smart cameras” without reference to AI may not have been captured.

*A second concern is that the criteria used by the author to consider that a city should be considered European is not clear. I was somewhat surprised to see deployments in the UK and Russia included in the results. The author mentioned using a list of cities adapted from Karam et al. Perhaps a clearer explanation on the nature of this adaption would be useful. I note that the spreadsheet provided by Karam et al groups together cities from Japan and Europe, and draws on a broad definition of Europe (again, including for example the UK and Russia). Although I will be very happy about seeing British projects included, I wonder if a focus on the European Union would be more appropriate for this publication.*

**Response: **This is a good point. Indeed, I should have clearly defined what counts as Europe. I have clarified this in the revised methodology section. This study uses the United Nations M49 classification (United Nations Statistics Division, n.d.) to determine which countries are included in Europe. The M49 classification is maintained by the United Nations Statistics Division and provides a standardized and internationally recognized grouping of countries and areas into geographical regions. Under this classification, the United Kingdom and the Russian Federation are included in Europe. I did consider restricting the analysis to European Union member states. However, this study aims to examine city-level AI deployments rather than programs that are specific to the institutional framework of the European Union. Defining the study area according to EU membership would introduce a political/institutional boundary. Also, the European Union is much narrower than geographical Europe (27 member states vs 44 countries). It would exclude many European countries, such as the United Kingdom, Norway, and Switzerland. I think the UN M49 classification is more appropriate because it provides a consistent scope that is independent of changes in EU membership.

*As much of the methodology for this study relies on automation, I hope code can be adapted and queries can be re-run with relative ease, although I realise that the results and conclusions will have to be adjusted.*

**Response: **Yes. The search is fully scripted. I have updated everything in the revised manuscript.

*I have some minor concerns in addition to the above, which are very minor, but I will mention for the sake of completeness. - "I identified 88 AI deployments" should probably be rephrased to avoid "I". Perhaps "The analysis identified"? "It shows a clear acceleration in adoption...". I am not sure what "it" refers to. (But it can refer to the analysis, if the change I suggested is implemented.*

**Response: **Thanks for pointing this out. I changed the wording to “the analysis” as suggested.

*The point of some of the examples mentioned in the introduction is not clear. Paragraphs 2 and 3, which discuss Noaen et al, Zemmouchi-Ghomari and Debbaghi et al feel like random examples, with no clear sense of why they were included. Are these major projects, or particularly illustrative?*

**Response: **Thank you, this is a fair point. Noaeen et al. (2022) is cited as evidence that the gap between technical potential and real-world deployment is acknowledged within the technical literature itself, while Zemmouchi-Ghomari (2025) and Debbaghi et al. (2025) are cited to illustrate the scale at which empirical work on actual deployments is currently conducted. I have made the argument clearer in the revised manuscript: The disconnect from real-world deployment is acknowledged within the technical literature itself. In a systematic review of reinforcement learning for network-scale traffic signal control, Noaeen et al. (2022) pointed out that many studies do not deal with or include real-world data, which challenges the validity of their results. … Large-scale empirical analyses across multiple cities and transportation modes are still rare. Two recent studies illustrate the scale at which such work is typically conducted. A case study of five cities on AI usage in Intelligent Transportation Systems found that … ( Zemmouchi-Ghomari, 2025). An evaluation of 12 shared automated vehicle pilots in Europe …  ( Debbaghi et al., 2025). Both studies are informative, but they cover a small number of sites, and neither establishes a baseline against which adoption can be assessed. Debbaghi et al. (2025) themselves call for standardized data collection processes and emphasize the need for baseline data for future deployments.

*Finally, I also that the authors discussion of year to year trends loses credibility because it was completed 9 months into 2025. A revision would provide an excellent opportunity to provide full data for that year, allowing for a more robust analysis of trends.*

**Response: ** This is a great idea, thank you! I have set the end date of the search to 31 December 2025, so the data and analysis now cover the full 2025. References European Commission. (2025). *Guidelines on the definition of an artificial intelligence system established by Regulation (EU) 20241689 (AI Act)*. https://digital-strategy.ec.europa.eu/en/library/commission-publishes-guidelines-ai-system-definition-facilitate-first-ai-acts-rules-application Premaratne, P., Jawad Kadhim, I., Blacklidge, R., & Lee, M. (2023). Comprehensive review on vehicle Detection, classification and counting on highways. *Neurocomputing*, *556*, 126627. https://doi.org/10.1016/j.neucom.2023.126627 United Nations Statistics Division. (n.d.). *Standard Country or Area Codes for Statistical Use (M49)*. Retrieved August 6, 2026, from https://unstats.un.org/unsd/methodology/m49/
